# Timing for green prescription: comparing the immediate and cumulative effects of different duration horticultural therapy programs on college students' mental health

**DOI:** 10.3389/fpsyg.2026.1838988

**Published:** 2026-06-05

**Authors:** Chang Liu, Lin Chen, Youlong Sun, Yidian Pan, Ruqi Chen

**Affiliations:** College of Landscape Architecture and Art, Fujian Agriculture and Forestry University, Fuzhou, Fujian, China

**Keywords:** college students, horticultural therapy, immediate and cumulative effects, intervention duration, mental health

## Abstract

**Background:**

Although the mental health benefits of horticultural therapy (HT) are well-established, the influence of intervention duration and the temporal dynamics of effects remain insufficiently explored, lacking evidence for optimal protocol design.

**Methods:**

This study employed a comparative experimental design with a short-duration HT program (4 sessions, *n* = 12) and a long-duration program (8 sessions, n=14), both conducted weekly. Immediate effects (mood states, affects, facial expressions, salivary cortisol) were measured before and after each session. Cumulative effects (meaning in life, depression, anxiety) were assessed at baseline, post-intervention, and at a 4-week follow-up. Non-parametric tests were used to analyze within-group changes and between-group differences in effect sizes.

**Results:**

For immediate effects, all psychological and physiological indicators showed significant improvement after each single session (|*r*| > 0.5, *p* < 0.05), with no significant difference in effect sizes between groups. For cumulative effects, the long-duration group demonstrated a significant and sustained increase in the presence of meaning in life (PML), which was maintained at the follow-up. While the short-duration group also showed a statistically insignificant trend of improvement in PML. Both programs significantly reduced depression levels immediately post-intervention, but these effects rebounded at follow-up. Anxiety decreased significantly only in the short-duration group post-intervention, with both groups showing a rebound at follow-up.

**Conclusion:**

HT activities provide stable and significant immediate mental restoration, unaffected by the overall program duration. While shorter programs are effective for transiently alleviating anxiety and depression, longer durations are crucial for fostering a stable and enduring sense of meaning in life. This study reveals a differentiated “dose-response” relationship for HT, providing refined evidence for designing time-sensitive intervention strategies tailored to specific mental health objectives.

## 1 Introduction

College students are in the transitional phase from adolescence to adulthood, with psychological functions and coping mechanisms that remain immature (Auerbach et al., 2016). When confronted with external stressors such as academic pressure, employment pressure, and relationship difficulties, they may experience significant psychological distress (Wilson et al., 2021; Chen et al., 2022, 2024), present higher risks for mental health issues and mental disorders (Auerbach et al., 2016; McGrath et al., 2023).

The World Mental Health International College Student Initiative (WMH-ICS) conducted a multinational cross-sectional survey from 2017 to 2023. Among a sample of 72,288 1-year university students from 77 universities across 18 countries, the positive screening rate for mental disorders in the 12 months preceding the survey was 57.4% (Mason et al., 2025). A report released by the American College Health Association in spring 2024 stated that 76.4% of college students nationwide experienced moderate to high levels of stress in the past 30 days, while 48.5% reported feeling lonely (ACHA, 2024). The Chinese National Mental Health Development Report (2021-2022) found that the depression detection rate among college students aged 15–26 (21.48%) was significantly higher than in other populations, with anxiety risk detection rates (45.28%) also elevated (Fu and Zhang, 2023).

Poor mental health among college students and the resulting adverse events not only threaten individual wellbeing, life safety, and family welfare (Mason et al., 2025),but also generate broader societal impacts through rapid public discourse dissemination, potentially destabilizing social structures (Zhang, 2019). Therefore, prioritizing the prevention and management of mental health risks among college students has become a global consensus in higher education. In China, most universities have established psychological crisis early warning mechanisms, implementing tiered interventions for students based on their risk levels (Zhang and Wang, 2018; Zhang, 2019; Zhao and Zuo, 2024). They are also actively exploring ways to integrate more diverse therapeutic activities into traditional counseling to reduce the impact of stigma (Zhang, 2019; Li et al., 2022b), thereby attracting more college students to participate and benefit from these services.

It is widely recognized that humans possess an innate affinity for nature (Wilson, 1984). Ulrich published a significant finding in Science magazine demonstrating that visual exposure to natural window views accelerates recovery after gallbladder surgery, and proposed the Stress Recovery Theory, which holds that natural environments help alleviate stress levels (Ulrich, 1984). Based on long-duration natural observations, Kaplan proposed the Attention Restoration Theory, which suggests that natural environments induce shifts in attention patterns and thus aid attention recovery (Kaplan and Kaplan, 1989). In recent years, a growing body of research has confirmed that exposure to nature offers numerous health benefits, including mood regulation, stress reduction, cognitive function restoration, and autonomic nervous system modulation (Hyvönen et al., 2023; Jiang et al., 2025).

Green Prescriptions are health promotion programs that utilize nature-based encounters as a systematic approach to prescription and implementation (Adewuyi et al., 2023). As early as 1699, The British Garden magazine documented the benefits of gardening for physical and mental health. In 1792, York Asylum in England incorporated playing with rabbits and chickens into its treatment programs. By the early 19th century, psychiatrists in northern Scotland prescribed “working on farms” as therapy. By the mid-19th century, British mental hospitals had patients cultivating plants in hospital gardens to alleviate their conditions (Li, 2000). In 1998, New Zealand's Ministry of Health formally proposed and spearheaded the “Green Prescription” initiative, encouraging doctors or health advisors to prescribe personalized outdoor activity recommendations based on patients' health conditions as an alternative or adjunct to traditional medication. Subsequently, countries including the UK, US, and Canada rolled out similar programs, integrating green prescriptions like horticultural therapy (HT), forest therapy, wilderness therapy, and climatotherapy into public health systems (CGT, 2025).

As a quintessential green prescription, HT refers to the practice of utilizing plants and gardening activities under the guidance of horticultural therapists to regulate the physiological, psychological, spiritual, and social health states of specific populations (AHTA). Due to its operational flexibility, HT has gained widespread adoption globally. Research confirms that HT improves mood (Wei et al., 2020), enhances wellbeing (Kor et al., 2025), alleviates anxiety (Joubert et al., 2024), mitigates depressive symptoms (Chen et al., 2025), and regulates autonomic nervous system activity (Guo et al., 2024) among various populations including the elderly (Wang et al., 2022), individuals with cognitive impairment (Wang et al., 2024),those with depression (Xu et al., 2023), veterans (Kelley et al., 2017), individuals with schizophrenia (Oh et al., 2018), and at-risk youth (Park et al., 2022).

In recent years, the impact of HT on college students has also drawn attention. A study indicated that just 5 min of HT activities (watering, pruning) improved mood, attention, and brain activity among college students undergoing prolonged online learning during COVID-19 isolation, outperforming conventional entertainment like mobile gaming (Li et al., 2022a). Long-duration HT activities may further enhance more stable psychological structures among college students. For instance, a study indicated significant improvements in depression, stress levels, and belongingness among veteran college students after six HT sessions compared to a control group (Kelley et al., 2017). Another experiment involving 176 college students revealed that participating in a 9-week HT program significantly enhanced the “emotional dimension” and “transcendence dimension” of positive psychological qualities compared to the control group (Li et al., 2022b).

Duration, frequency, and other temporal characteristics are crucial components of green prescription dosing. However, the “time-dose-response” patterns of HT remain unclear, and there is insufficient evidence to determine the optimal duration of HT programs. In a 12-week, twice-weekly HT controlled trial involving post-stroke depression patients, no significant efficacy differences were observed between the HT group and the conventional therapy group by week 4. However, by week 12, the HT group demonstrated superior improvements across all measures of neurological function, activities of daily living, depressive symptoms, and anxiety symptoms compared to the control group (Su et al., 2021). A separate 8-week, five-times-weekly controlled trial of HT for individuals with depression yielded similar findings: no significant differences emerged between the HT and control groups at week 4, but by week 8, the therapy group demonstrated greater improvements in depressive symptoms, quality of life, and social functioning compared to the control group (Wu et al., 2018). Duration and frequency may play distinct roles in shaping intervention outcomes. For elderly women with mild cognitive impairment, a low-frequency, long-duration program (once weekly for 15 weeks) proved more effective than a high-frequency, short-duration program (twice weekly for 7.5 weeks) in maintaining neurocognitive function and improving life satisfaction, though the high-frequency group showed greater efficacy in alleviating depressive symptoms (Kim et al., 2020).

Although empirical studies using duration as a primary variable are scarce, meta-analytic subgroup comparisons reveal some influence of duration characteristics on intervention outcomes. Compared to longer durations, 4–8 week HT programs showed greater efficacy in improving depressive mood among older adults (Xu et al., 2023). Another study suggested that 5–8 weeks may represent the optimal duration for HT programs targeting depressive symptom improvement (Chen et al., 2025). However, enhancing cognitive function in elderly individuals with dementia may require programs lasting 6 months or longer (Wang et al., 2024).

Additionally, the effects of HT may also be time-sensitive. Certain indicators—such as physiological arousal levels, autonomic nervous system activity, and emotional states—can demonstrate noticeable immediate changes following a single session. For instance, studies found that single-session activities like seed sowing, thinning seedlings, and arranging succulent plant combinations significantly reduced heart rate and alleviated physiological stress in isolated elderly individuals. Seed sowing and thinning activities also markedly enhanced positive emotions and facial expressions of pleasure while decreasing negative emotions (Wei et al., 2020). Exposure to a laboratory environment featuring natural soundscapes and plant aromas for just 10 min significantly reduced physiological stress levels and improved emotional states (Guan et al., 2025). Another study found that merely 2 min of visual exposure to a virtual natural environment improved emotional states associated with cognitive fatigue (Mihara et al., 2023). However, deeper psychological structures may require cumulative improvements achieved through repeated activities over longer periods. For instance, a 4-week “virtual healing garden” intervention significantly reduced depression, anxiety, and stress levels in elderly women, with effects persisting 2 weeks after the program concluded (Szczepanska-Gieracha et al., 2021). Yet current research rarely measures and distinguishes immediate and cumulative effects within the same framework, leaving evaluations of HT's efficacy lacking temporal dimensions.

To reveal the impact of duration on the effectiveness of HT psychological interventions and to distinguish the temporal scale of effects, this study designed HT programs with varying intervention durations for college students. It measured both the immediate effects of single sessions and the cumulative effects of the entire program to address the following questions: (1) Does the immediate effect of a single HT session change with variations in duration? (2) Do cumulative effects of the HT program (including from baseline to post-intervention, baseline to follow-up, and post-intervention to follow-up) differ across treatment groups with varying durations?

## 2 Method

### 2.1 Group and intervention

Two groups of HT programs with different durations were established. The short-duration group comprised four therapy activities: arranging flowers, breathing exercises in nature, smelling the scents of plants, and transplanting potted plants. Each activity was conducted weekly over four consecutive weeks. The long-duration group comprised eight sessions: the first and fifth sessions involved arranging flowers; the second and sixth sessions featured breathing exercises in nature; the third and seventh sessions focused on smelling the scents of plants; and the fourth and eighth sessions involved transplanting potted plants. Each activity was conducted once per week over 8 weeks. Arranging flowers referred to the art of creating decorative pieces with natural beauty by skillfully arranging plant parts such as flowers, leaves, branches, and fruits. Breathing exercises in nature involved actively controlling breathing rhythm, depth, and pattern through simple physical movements and nature-based meditation in a garden setting. Smelling the scents of plants comprised three components: smelling plant scents, memorizing aromas, and crafting perfumes. Transplanting potted plants involved planting multiple succulent species alongside decorative elements like pots and mulch stones in a single container according to aesthetic principles. Before and after each session, participants completed indicator questionnaires and provided saliva samples for cortisol analysis as pre- and post-test data. Each activity lasted approximately 40 min, with pre- and post-assessments taking about 15 min.

The study focused on examining the impact of overall intervention duration on the effects of HT after the intervention had ended, as well as exploring the maintenance effects associated with that duration. Therefore, a between-group design with two independent groups was adopted. The two groups were assessed under identical activity timing and venue settings to hold external conditions constant.

**Figure 1:**
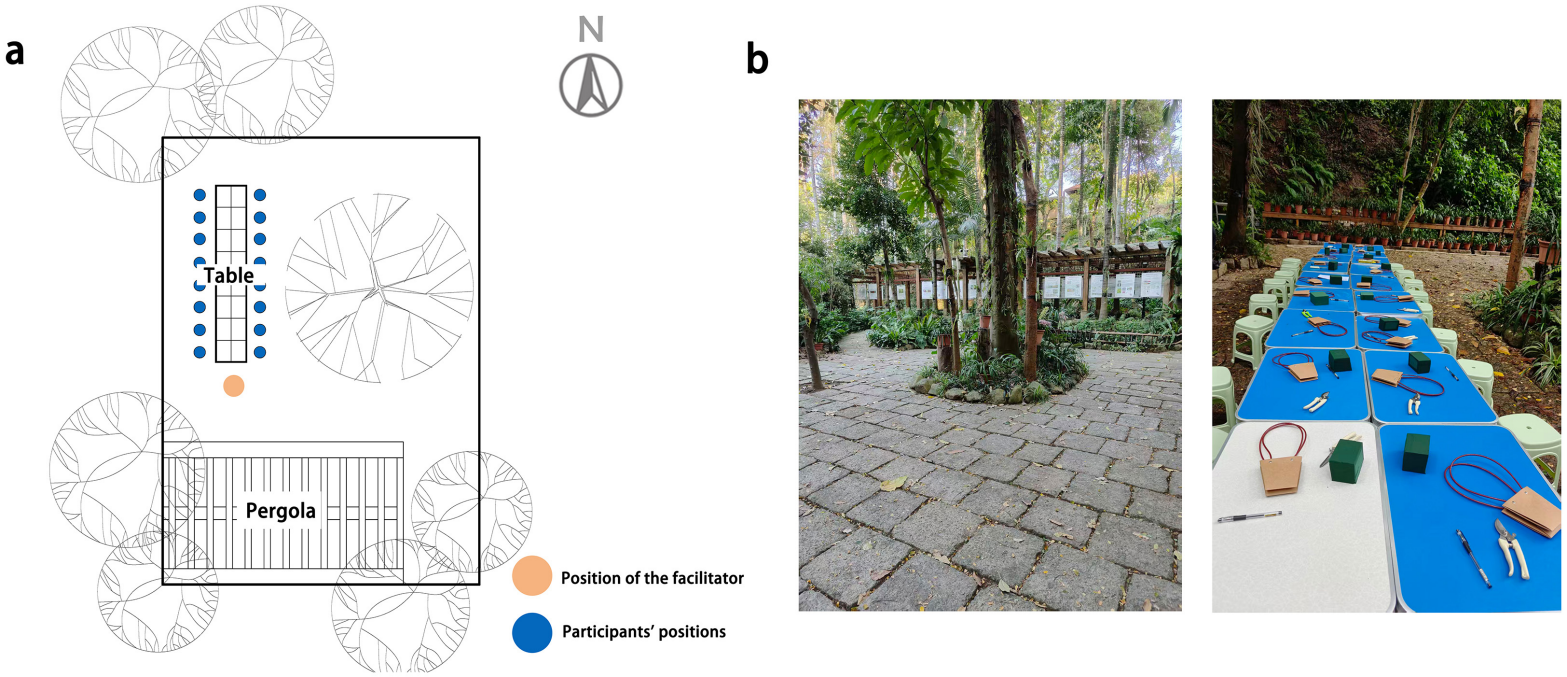
Diagram of the activity venue. **(a)** Floor plan of the activity venue; **(b)** live scene of the activity venue.

Each session was staffed by one trained facilitator and one assistant. All activities took place between April and June 2025 at an outdoor practice base of Fujian Agriculture and Forestry University. The rectangular site, surrounded by abundant vegetation, featured a stone-paved plaza and a wooden pergola (Figure 1).

The temperature range during the activity periods was managed using real-time weather forecasts; activities were postponed by 1 day in the event of rain or high temperatures. Across all experimental sessions, to ensure consistency of salivary cortisol levels, activities were scheduled between 14:00 and 16:00. The conditions during these sessions were maintained within a temperature range of 21 °C to 27 °C, humidity between 60% and 75%, and an absence of noticeable noise at the activity venue.

### 2.2 Participants

In March 2025, participants were recruited from college students via social platforms (QQ, WeChat, etc.) and screened based on the following criteria:

(1) No pollen allergies;(2) No physical disabilities or motor impairments;(3) No sensory impairments, such as color blindness, color weakness, hearing impairment, or olfactory dysfunction;(4) The total score on the Symptom Checklist-90 (SCL-90) was between 70 and 129, with subscale scores for anxiety and depression > 0(Rating from 0 to 4 levels) (Derogatis, 1977), indicating a certain tendency toward mild to moderate psychological distress (Chen and Li, 2003).

After screening (Table 1), 12 participants were assigned to the short-duration group (age 22.50 ± 2.07 years; 4 males [33.3%] and 8 females [66.7%]; 8 masters and 4 bachelors), and 14 participants were assigned to the long-duration group (age 22.21 ± 2.04 years; 7 males and 7 females, 50.0% each; 9 masters and 5 bachelors). Participants were randomly allocated to groups using a computer-generated random sequence.

**Table 1:** Demographic characteristics.

Variable	Short-duration	Long-duration	Statistic	*df*	*p*
Age, mean (SD)	22.5 (2.07)	22.21 (2.04)	*t* = 0.35	24	0.727
Gender, *n* (%)					
Male	4 (33.3%)	7 (50%)			
Female	8 (66.7%)	7 (50%)	χ^2^ = 0.74	1	0.391
Education level, *n* (%)					
Bachelor	4 (33.3%)	5 (35.7%)			
Master	8 (66.7%)	9 (64.3%)	χ^2^ = 0.02	1	0.899

The study was conducted in accordance with the Declaration of Helsinki and the Guiding Principles for Ethical Research Involving Human Subjects issued by the Ministry of Education of the People's Republic of China. All experimental protocols were reviewed and approved by College of Landscape Architecture and Art, Fujian Agriculture and Forestry University. All participants voluntarily signed informed consent forms prior to the experiment.

### 2.3 Indicators and measurements

#### 2.3.1 Immediate effects

The following indicators were measured before (pre-test) and after (post-test) each activity:

Total Mood Disturbance (TMD): TMD was measured using the Chinese translation of McNair's Brief Profile of Mood States (McNair and Droppleman, 1992; Chi and Lin, 2003). This 5-point Likert scale (0–4 points) comprises 30 items across five dimensions: tension, anger, fatigue, depression-confusion, and energy. It includes six reverse-scored items, with the total sum reflecting participants' current overall mood disturbance level.

Positive Affect (PA) and Negative Affect (NA): PA and NA were measured using the Chinese translation of the PA and NA Scale (Watson et al., 1988; Li et al., 2003). This 5-point Likert scale (1–5 points) comprises two dimensions—PA and NA—each with 10 items, yielding separate total scores for each dimension.

Facial expression (FE) was measured using the Faces Scale (Andrews and Withey, 1976). This scale consists of a single item: seven facial expressions ranging from an angry frown (strongest negative emotion) to a happy smile (strongest positive emotion), scored sequentially from 1 to 7.

Salivary Cortisol: Salivary cortisol (the free form of the glucocorticoid hormone cortisol secreted by the adrenal cortex's zona fasciculata) reflects the functional activity of the hypothalamic-pituitary-adrenal (HPA) axis, indicating the degree of physiological arousal (Hellhammer et al., 2009). Saliva samples were collected before and after each session for cortisol analysis. Due to the natural right-skewed distribution of salivary cortisol concentrations, values were log-transformed to better meet parametric statistical requirements (Miller and Plessow, 2013).

#### 2.3.2 Cumulative effects

The following measures were assessed before the first session commenced, immediately after the final session concluded, and at the 4th week post-activity completion:

Presence of Meaning in Life (PML) and Search for Meaning in Life (SML): PML and SML were measured using the Chinese translation of the Meaning in Life Questionnaire (Steger et al., 2006; Liu and Gan, 2010). This 7-point Likert-scale (1–7 points) instrument comprises two dimensions—PML and SML—each containing five items. Total scores were calculated for each dimension.

Anxiety and Depression: Anxiety and Depression were measured using the Chinese versions of the Anxiety and Depression subscales of the SCL-90 (Derogatis, 1977; Tang et al., 1999). The Anxiety Scale comprises 10 items, and the Depression Scale comprises 13 items. Both scales employ a 5-point Likert scale: “None” = 0 point, “Very mild” = 1 points, “Moderate” = 2 points, “Slightly severe” = 3 points, “Severe” = 4 points.

### 2.4 Data analysis

Prior to formal analysis, the Shapiro-Wilk test was used to assess the normality of outcome variable distributions. Independent samples *t*-tests and chi-square tests were employed to verify intergroup consistency of demographic characteristics. The Mann-Whitney *U* test was used to assess intergroup homogeneity of baseline outcome variables.

For immediate effects, the Wilcoxon signed-rank test was first applied to compare pre- and post-session measurements for each activity, with the effect size *r* calculated as Z/√N. To compare the magnitude of immediate effects between the short-duration and long-duration groups, the analysis focused on activity-level comparisons to preserve the pairing of session content. For each of the four activity types (Arranging Flowers, Breathing Exercises in Nature, Smelling the Scents of Plants, and Transplanting Potted Plants), the effect size from the short-duration group's single session was separately compared with that from the first occurrence and the second occurrence of the same activity in the long-duration group. Fisher's *Z*-transformation was applied to the *r* values to approximate normality and stabilize variance, and *Z*-tests were conducted to assess differences in effect magnitude. Additionally, the median *r* across all sessions was calculated for each group to provide a descriptive synthesis of the overall effect magnitude. This aggregate metric serves purely as a convergent summary rather than a standalone confirmatory test, given the repeated-measures structure of the sessions. All *p*-values from these comparisons were adjusted using the Benjamini-Hochberg FDR correction to control for multiplicity.

For cumulative effects, the Friedman test was first used to assess overall differences in indicator levels across the three time points (baseline, post-intervention, follow-up) within each group (short-duration and long-duration). Subsequently, the Wilcoxon signed-rank test was used to compare paired time points (baseline vs. post-intervention, baseline vs. follow-up, post-intervention vs. follow-up) within each group, with effect sizes (*r*) calculated. Finally, Fisher's *Z*-transformation was applied to the *r* values, and Z-tests were conducted to compare intergroup differences in effect sizes across indicators.

All statistical analyses were performed using RStudio (version 4.3.0). Packages employed included tidyverse, ggplot2, stats, rstatix, and effsize.

## 3 Results

### 3.1 Data distribution and sample consistency

The Shapiro–Wilk test indicated that depression, NA, PA, and FE scores were not normally distributed, whereas age was normally distributed. Independent sample *t*-test showed no difference in age between the two groups (*t* (24) = 0.35, *p* = 0.727). Chi-square test indicated no difference in gender (χ^2^ = 0.74, *p* = 0.391) and educational level (χ^2^ = 0.02, *p* = 0.899) between the two groups. Mann-Whitney U test demonstrated no difference in baseline values of all outcome measures between the two groups (all *p* > 0.05).

### 3.2 Immediate effects

#### 3.2.1 Pre-test vs. post-test

**Figure 2:**
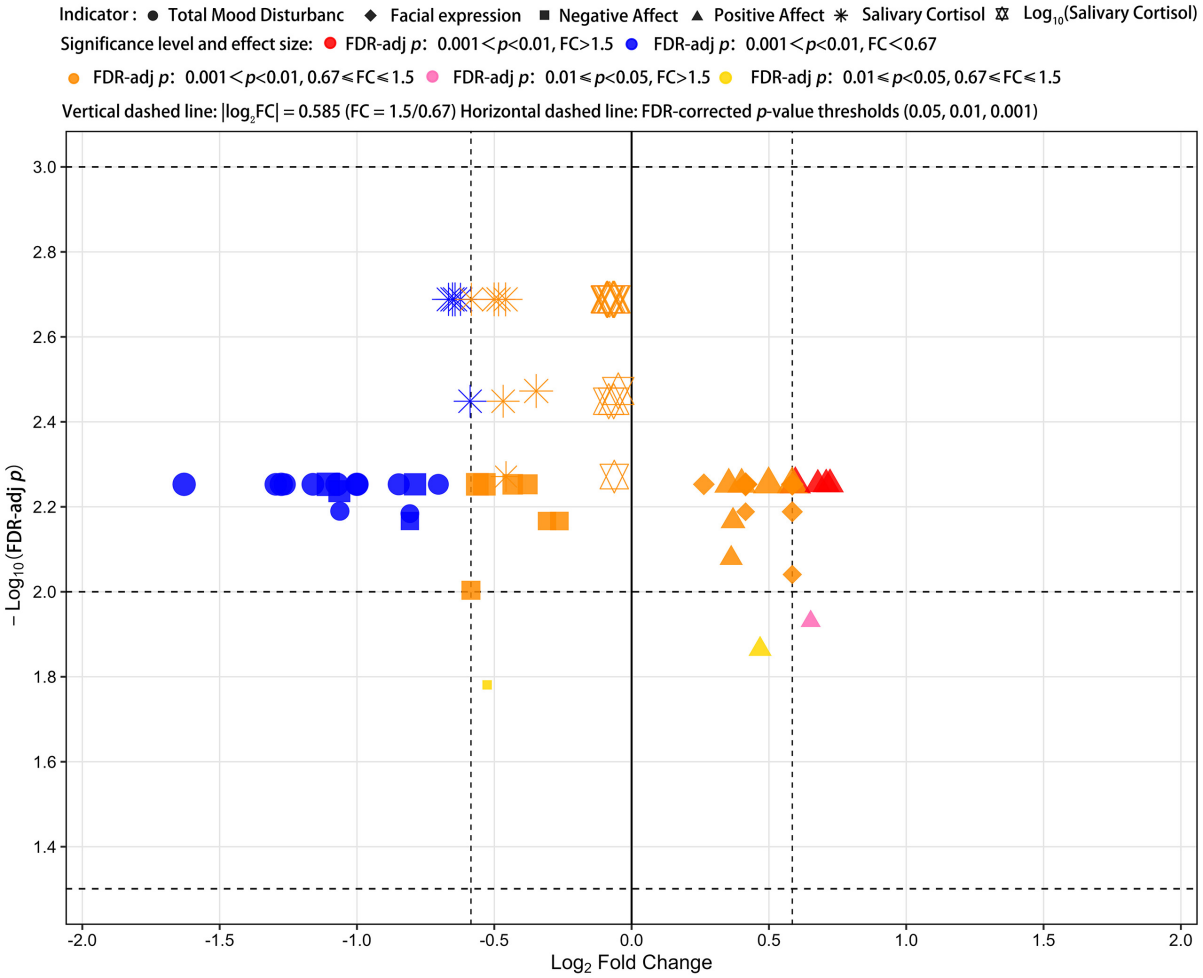
Differences and significance of pre- and post-test measures for the immediate effect indicator.

A paired-sample Wilcoxon signed-rank test was conducted for all 12 session-level pre-post comparisons across the five immediate-effect indicators. After FDR correction, *p*-values ranged from 0.001 to 0.017, indicating statistically significant improvements after every single HT session, regardless of activity type or intervention duration (Figure 2). Specifically, TMD decreased significantly; FE scores increased significantly; NA dropped, and PA increased; both raw and log10-transformed salivary cortisol concentrations decreased significantly after each session. All effect sizes (*r*) exceeded |0.50|, indicating large immediate effects.

#### 3.2.2 Intergroup differences in effect size

**Figure 3:**
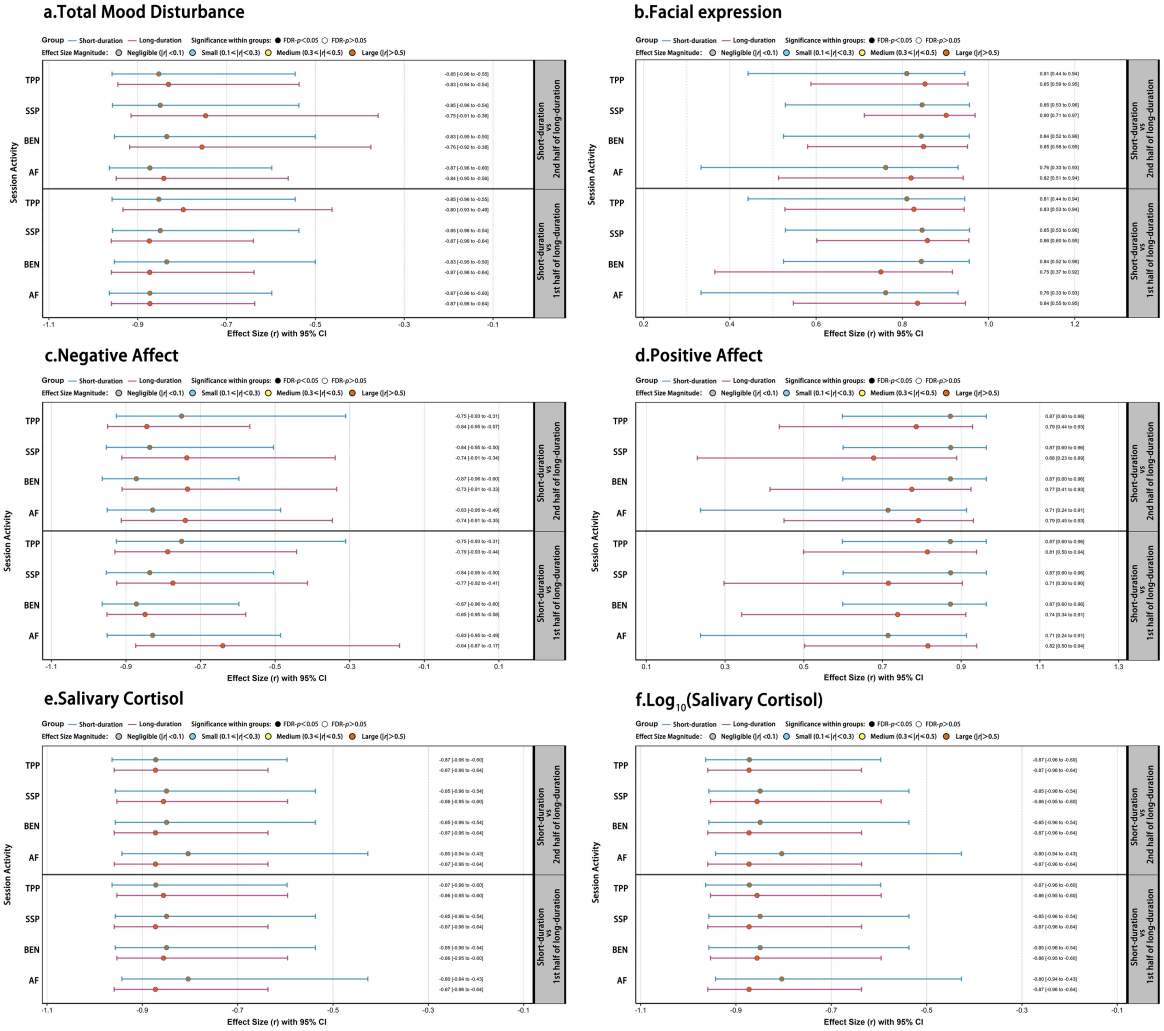
Forest plot of between-group comparisons of effect sizes for immediate effect indicators. AF = Arranging Flowers; BEN = Breathing Exercises in Nature; SSP = Smelling the Scents of Plants; TPP = Transplanting Potted Plants.

For all sessions, the *r* values for TMD ranged from −0.75 to −0.87, those for FE ranged from 0.75 to 0.90, those for NA ranged from −0.64 to −0.87, those for PA ranged from 0.68 to 0.87, and those for Salivary Cortisol ranged from −0.80 to −0.87, and Log_10_ Salivary Cortisol ranged from −0.80 to −0.87. All indicators had |*r*| > 0.5, indicating large effect sizes (Figure 3). The overall median effect sizes were descriptively similar between groups (Table 2).

**Table 2:** Intergroup comparisons of effect sizes for immediate and cumulative effects between long-duration and short-duration groups.

Effect		Indicator	***R***		*Z-diff*	*p*	*FDR-adj p*
			Short-duration	Long-duration			
Immediate Effect		TMD	−0.85	−0.84	-	-	-
		FE	0.83	0.84	-	-	-
		NA	−0.83	−0.76	-	-	-
		PA	0.87	0.78	-	-	-
		Salivary cortisol	−0.85	−0.87	-	-	-
		Log_10_ salivary cortisol	−0.85	−0.87	-	-	-
Cumulative effect	Baseline-post-intervention	PML	0.40	0.79	1.46	0.145	0.818
		SML	0.16	0.42	0.65	0.518	0.818
		Anxiety	−0.84	−0.58	1.25	0.211	0.818
		Depression	−0.78	−0.66	0.58	0.564	0.818
	Baseline-follow-up	PML	0.48	0.73	0.92	0.356	0.818
		SML	0.16	0.45	0.73	0.467	0.818
		Anxiety	−0.25	−0.35	−0.25	0.800	0.818
		Depression	−0.41	−0.54	−0.37	0.710	0.818
	Post-intervention-follow-up	PML	0.26	0.05	−0.49	0.624	0.818
		SML	0.00	0.10	0.23	0.818	0.818
		Anxiety	0.27	−0.13	−0.91	0.362	0.818
		Depression	0.28	0.53	0.66	0.507	0.818

*Z*-diff is the *Z*-score for the difference between Fisher's *Z*-transformed effect sizes of the long-duration and short-duration groups. For immediate effects, the reported *r* values represent the median effect size across all sessions for each group. As a descriptive summary, no aggregate statistical test (*Z* or *p*-value) is reported here to avoid pseudo-replication due to repeated measures within groups; formal between-group comparisons at the session level are presented in Section 3.2.2 and Figure 3. For cumulative effects, effect sizes were compared across 4 indicators × 3 time contrasts (12 independent tests), and the reported FDR-adj *p* values were obtained using the Benjamini–Hochberg procedure applied to this family of 12 comparisons.

For every activity type, the effect size from the short-duration group's single session was compared with both the first and second occurrences of the same activity in the long-duration group. No significant differences were observed in any of these activity-specific, content-matched comparisons (all FDR-adjusted *p* > 0.05; Figure 3), suggesting that the magnitude of immediate improvement was independent of whether the activity was experienced for the first time or repeated within a longer program. A supplementary descriptive summary using the median *r* of all sessions indicated a similar pattern, with highly comparable effect sizes between groups across all indicators (Table 2, upper panel). The descriptive consistency between the median effect sizes (Table 2) and the formal session-level comparisons (Figure 3) strengthens the conclusion that immediate restorative effects remain stable regardless of total program duration.

### 3.3 Cumulative effects

#### 3.3.1 Variation among baseline, post-intervention and follow-up

From baseline to post-intervention and then to follow-up, the trend in cumulative effect indicators showed high consistency across both groups.

**Figure 4:**
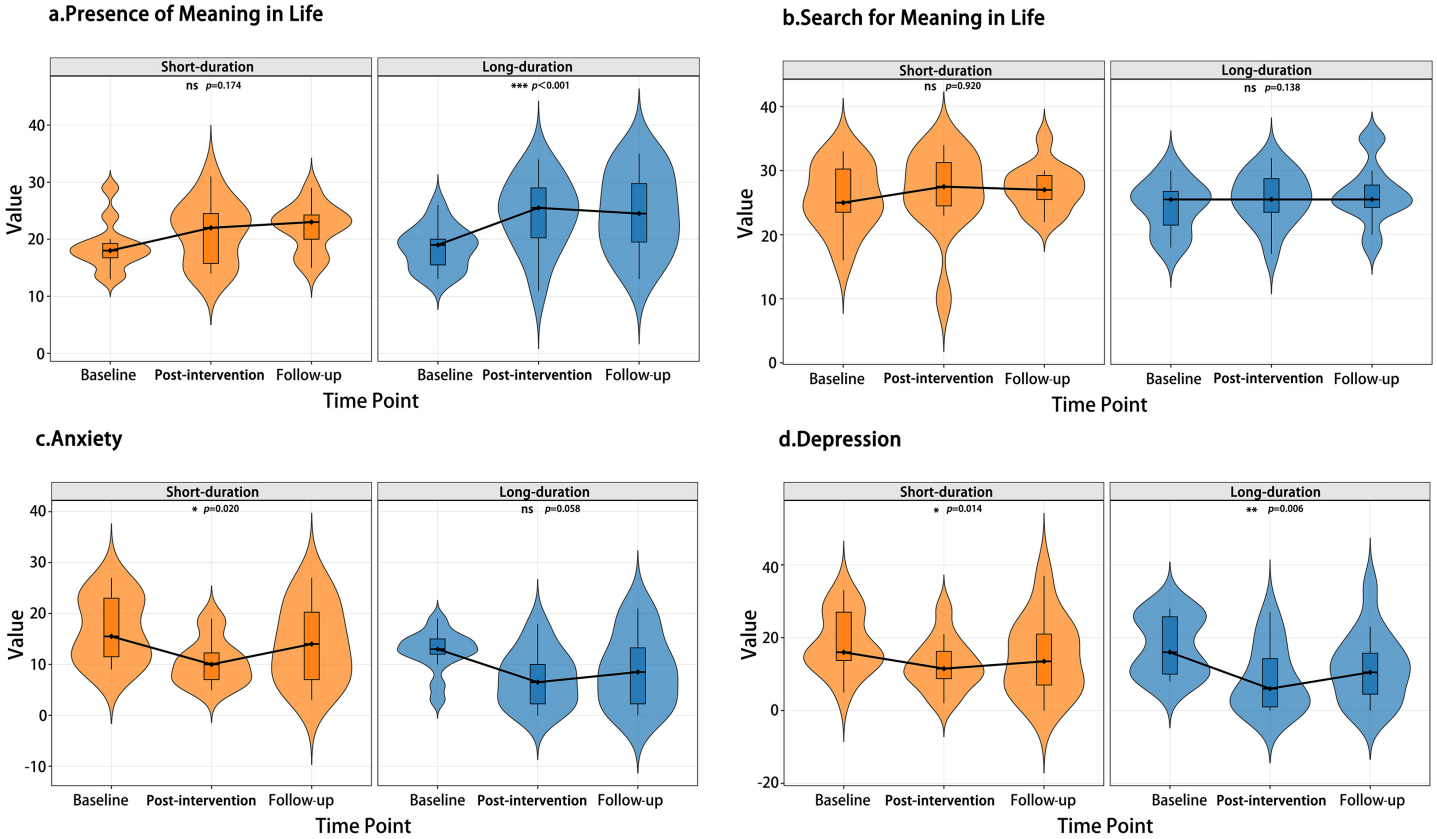
Value distribution of cumulative effect indicators. The significance level in the figure legend corresponds to the results of the Friedman test.

**Figure 5:**
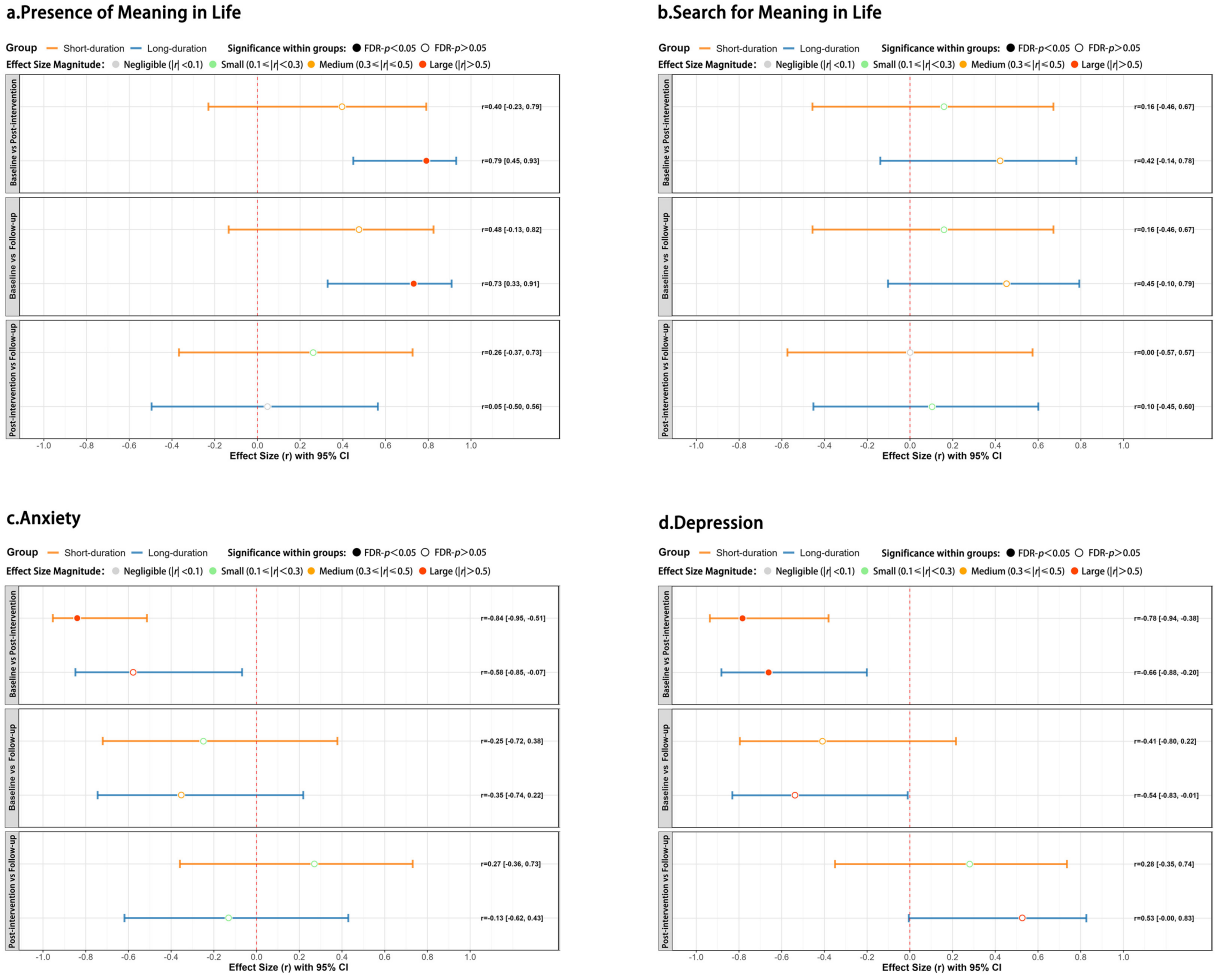
Forest plot comparing effect sizes of cumulative effect measures. Error bars represent 95% confidence intervals. No statistically significant between-group differences were observed for any indicator between the short-duration and long-duration groups.

For PML, both groups showed an increasing trend that stabilized after the intervention (Figure 4a). As shown in Table 3 and Figure 5a, in the short-duration group, median PML rose from 18.00 (IQR = 16.75–19.25) at baseline to 22.00 (15.75–24.50) post-intervention and remained at 23.00 (20.00–24.25) at follow-up; however, the overall change was not statistically significant (χ^2^ (2) = 3.500, *p* = 0.174). In the long-duration group, PML increased significantly from 19.00 (15.50–20.00) at baseline to 25.50 (20.25–29.00) post-intervention and was maintained at 24.50 (19.50–29.75) at follow-up (χ^2^ (2) = 15.269, *p* < 0.001). *Post-hoc* pairwise Wilcoxon tests confirmed significant baseline-to-post-intervention (*r* = 0.79) and baseline-to-follow-up (*r* = 0.73) differences in long-duration group.

**Table 3:** Levels and differences in cumulative effect indicators at baseline, post-intervention, and follow-up.

Group	Indicator	Median(IQR)			*df*	*χ^2^*	*p*
		Baseline	Post-intervention	Follow-up			
Short-duration	PML	18.00 (16.75, 19.25)	22.00 (15.75, 24.50)	23.00 (20.00, 24.25)	2	3.500	0.174
	SML	25.00 (23.50, 30.25)	27.50 (24.50, 31.25)	27.00 (25.50, 29.25)	2	0.167	0.920
	Anxiety	15.50 (11.50, 23.00)	10.00 (7.00, 12.25)	14.00 (7.00, 20.25)	2	7.787	**0.020**
	Depression	16.00 (13.75, 27.00)	11.50 (8.75, 16.25)	13.50 (7.00, 21.00)	2	8.522	**0.014**
Long-duration	PML	19.00 (15.50, 20.00)	25.50 (20.25, 29.00)	24.50 (19.50, 29.75)	2	15.269	**<** **0.001**
	SML	25.50 (21.50, 26.75)	25.50 (23.50, 28.75)	25.50 (24.25, 27.75)	2	3.962	0.138
	Anxiety	13.00 (12.00, 15.00)	6.50 (2.25, 10.00)	8.50 (2.25, 13.25)	2	5.698	0.058
	Depression	16.00 (10.00, 25.75)	6.00 (1.00, 14.25)	10.50 (4.50, 15.75)	2	10.302	**0.006**

All data are presented as median (interquartile range). Bold values indicate that *p* < 0.05.

For SML, scores remained largely stable across the three time points (Figure 4b). Neither the short-duration group (χ^2^ (2) = 0.167, *p* = 0.920), nor the long-duration group (χ^2^ (2) = 3.962, *p* = 0.138) exhibited significant change (Table 3).

Anxiety exhibited a pattern of initial decline followed by rebound (Figure 4c). As shown in Table 3 and Figure 5c, in the short-duration group, median anxiety scores decreased significantly from 15.50 (IQR = 11.50–23.00) at baseline to 10.00 (7.00–12.25) post-intervention and then rebounded to 14.00 (7.00–20.25) at follow-up (χ^2^ (2) = 7.787, *p* = 0.020). The baseline-to-post-intervention reduction was large and significant (*r* = −0.84), whereas the baseline-to-follow-up difference was small and non-significant (*r* = −0.25). The long-duration group showed a similar but statistically weaker pattern: anxiety dropped from 13.00 (12.00–15.00) to 6.50 (2.25–10.00) and rebounded to 8.50 (2.25–13.25), with the Friedman test failing to reach conventional significance (χ^2^ (2) = 5.698, *p* =0.058).

Depression exhibited a pattern of initial decline followed by a slight rebound (Figure 4d). As shown in Table 3 and Figure 5d, In the short-duration group, median scores fell from 16.00 (IQR = 13.75–27.00) to 11.50 (8.75–16.25) and then rose to 13.50 (7.00–21.00) at follow-up, (χ^2^ (2) = 8.522, *p* = 0.014). In the long-duration group, scores fell from 16.00 (10.00–25.75) to 6.00 (1.00–14.25) and rebounded to 10.50 (4.50–15.75), χ^2^ (2) =10.302, *p*=0.006. In both cases, the baseline-to-post-intervention reductions were large and significant (*r*= −0.78 and −0.66, respectively), while baseline-to-follow-up differences were moderate and not statistically significant after correction.

#### 3.3.2 Intergroup differences in effect sizes

Between-group comparisons of cumulative effect sizes (Fisher's *Z*) revealed no statistically significant differences for any indicator at any time contrast (all FDR-adjusted *p*>0.05;Table 2, lower panel, and Figure 5). Numerically, the short-duration group showed a larger post-intervention anxiety reduction (*r* = −0.84) than the long-duration group (*r* = −0.58), but this difference was not significant (*Z* = 1.25, FDR-adjusted *p* = 0.818). For PML, the long-duration group's large gain (*r* = 0.79) was not statistically distinguishable from the short-duration group's moderate increase (*r* = 0.40; *Z* = 1.46, FDR-adjusted *p* = 0.818).

## 4 Discussion

### 4.1 Immediate effect indicators

#### 4.1.1 Immediate effects are significant and large

Non-parametric test results indicated that participants demonstrated significant improvements in psychological indicators (TMD, FE, NA, PA) following a single HT intervention, regardless of activity duration or type. It was confirmed that HT possesses a distinct immediate psychological recovery effect. Concurrently, salivary cortisol concentrations decreased significantly post-activity, further supporting that HT activities significantly suppress HPA axis activity and induce a physiologically relaxed state (Qiu et al., 2023; Li et al., 2025). While the reduction in cortisol is consistent with enhanced parasympathetic tone observed in nature-exposure studies (e.g., increased heart rate variability), the present study solely assessed HPA-axis output.

Previous studies have repeatedly demonstrated that even a single HT session can yield significant improvements. Jung found that brief exposure to biophilic design elements—such as green walls—even in virtual reality settings, significantly increased alpha wave activity and improved mood (Jung et al., 2023). Another study reported that just 5 min of plant aroma exposure significantly enhanced psychological and physiological comfort among university students (Zhang et al., 2025). Collectively, these findings underscore HT 's strong intervention potential for mental health.

This discovery suggests that the human sensory system has high adaptability to nature. Having evolved in natural environments, human sensory systems are better attuned to natural settings or elements than built environments (Segundo-Ortin and Raja, 2024). Consequently, natural exposure rapidly alleviates stress levels and improves mood without requiring extensive cognitive processing (Wilson, 1984; Kaplan and Kaplan, 1989). Previous research further substantiated this conclusion, demonstrating that just 10 min of nature exposure can enhance positive emotions while reducing negative emotional indicators (Guan et al., 2025). Furthermore, HT activities are predominantly characterized by low-intensity and clear objectives, allowing participants to receive immediate positive feedback and a sense of control through simple actions. Additionally, these activities simultaneously stimulate multiple sensory channels—visual, auditory, olfactory, and tactile—yielding greater therapeutic efficacy than methods relying on single-sensory stimulation.

#### 4.1.2 Immediate effects do not change with extended duration

As duration increases, the immediate effects of repeated participation in a given activity type show no significant difference from those of initial participation. That is, these effects neither increase nor decrease, but remain at a relatively stable level.

This indicates that the immediate psychological recovery effects of HT remain stable at a weekly intervention frequency. This finding may relate to the balance between the “learning effect” and the “fatigue effect” (Anderson, 1980): As participants become more familiar with the activity content and group environment, their sense of control and willingness to participate increase, thereby counteracting any potential decline in interest due to repeated participation. A meta-analysis has also supported that 1–2 sessions per week is one of the optimal frequencies for HT programs (Chen et al., 2025).

### 4.2 Cumulative effect indicators

#### 4.2.1 Presence of meaning in life and search for meaning in life

Regarding PML, statistically significant improvements in PML levels were observed at the conclusion of the intervention program in the long-duration group. The short-duration group also demonstrated a trend toward improvement, with these gains persisting 4weeks post-intervention.

Although life meaning is rarely discussed in current HT research, “establishing a life connection between humans and plants, and using plant life to influence human life” is considered a core mechanism of HT (Li et al., 2023). Meaning in Life represents an individual's deep, relatively stable cognitive structure regarding life's purpose and value, whose alteration typically requires more profound and prolonged interventions (Steger et al., 2006). Consequently, the improvement in PML was more pronounced and robust in the long-duration group. However, SML was unaffected by the HT program. This may stem from HT being an “experiential” intervention rather than “cognitive” instruction. Participants directly “experience” feelings of fulfillment, calmness, connection, and accomplishment through hands-on gardening activities (such as sowing, watering, pruning), rather than having their motivation to seek meaning in life stimulated through didactic teaching.

#### 4.2.2 Anxiety and depression

Following the short-duration HT program, participants' anxiety levels showed significant improvement; the long-duration group also exhibited a trend toward improvement. One study combining EEG with virtual reality technology demonstrated that subjects exposed to plant walls and digital natural landscapes in a hospital setting exhibited significantly reduced State-Trait Anxiety Inventory scores (Jung et al., 2023). Another study involving 12 HT sessions for women demonstrated statistically significant reductions in both state and trait anxiety scores in the experimental group compared to the control group (Kim and Park, 2018). This aligns with the findings of this study. The essence of anxiety lies in a sense of loss of control over the future. HT activities involve clear, simple tasks where participants receive immediate, tangible positive feedback after completing each action, rapidly rebuilding a sense of control over their environment.

Following both short-duration and long-duration HT programs, participants demonstrated significant improvements in depression levels. A pilot study indicated that a 6-week garden walking program (either independent or guided) significantly alleviated depressive symptoms among community-dwelling older adults (McCaffrey et al., 2011). Another study demonstrated that a one-week regimen of approximately 20 min daily of nature therapy (incorporating sensory immersion, deep breathing, and meditation) significantly alleviated depressive symptoms among young adults during the pandemic (Sundara Rajoo et al., 2021), further validating HT 's therapeutic efficacy for depression. The essence of depression lies in feelings of loss regarding the past, a sense of worthlessness in the present, and hopelessness about the future. HT activities visualize a sense of accomplishment through a series of simple, concrete, and achievable micro-tasks. This directly counters the feelings of worthlessness and hopelessness associated with depression, rebuilding hope through witnessing the growth of life.

Extending the duration does not amplify improvements in anxiety or depression; in fact, it may even diminish them, suggesting diminishing marginal returns. The novelty and strong motivation during the initial phase likely peaked marginal benefits within the first four sessions. Subsequent sessions may consolidate gains but struggle to achieve further breakthroughs. This finding aligns with research showing comparable mindfulness gains between 4-week and 8-week programs for non-clinical populations (Demarzo et al., 2017).

Following the intervention program's conclusion, anxiety and depression levels rebounded, indicating that the improvements were not effectively sustained. Without external reinforcement and ongoing support, HT struggles to automatically translate into individuals' intrinsic, enduring emotional regulation capabilities. According to emotion regulation theory, long-duration maintenance of benefits may require interventions incorporating more cognitive restructuring, self-monitoring, or environmental support components (Gross, 2015).

#### 4.2.3 Immediate–cumulative effect interplay

The above results reveal distinct temporal dynamics in the immediate and cumulative effects of HT. The immediate psychological restorative effect of a single session remained stable and did not attenuate with an extended intervention duration. In contrast, emotional symptoms such as anxiety and depression, although quickly alleviated in the short term, rebounded once the intervention concluded. A stable and enduring enhancement in the sense of meaning in life, however, required a longer intervention period. These divergent patterns can be understood through a dual-pathway framework of HT. Immediate effects are primarily underpinned by a bottom-up sensory–affective pathway: individuals generate rapid and automatic restorative responses to natural environments and simple, achievable tasks (Ulrich, 1984). At a weekly frequency, these responses remain largely invariant, thereby providing participants with a consistent and stable buffer against stress. Cumulative psychological changes, on the other hand, necessitate repeated experiential engagement to gradually construct higher-order cognitive structures (such as meaning in life). Once established, this cognitive-level restructuring may partially protect against emotional rebound (Steger et al., 2006; Gross, 2015). It is plausible that the reliable emotional improvement delivered by each session provides the foundation for cumulative, deeper-level cognitive restructuring (Stumpp et al., 2024), although this mechanistic pathway was not directly tested in the present study.

### 4.3 Implications

This study reaffirms the efficacy of HT for college students, demonstrating significant outcomes within this population and providing clear evidence for its broader implementation.

Regarding immediate improvement, extending the duration of HT sessions at a reasonable frequency yields comparable results to multiple shorter sessions. HT can serve as a routine component of campus mental health prevention and treatment programs for college students, rather than being confined to single experiential interventions.

Regarding cumulative effects, both the 4-week and 8-week programs led to within-group reductions in anxiety and depression, with the between-group difference in effect size not reaching statistical significance. Given that the shorter intervention achieved comparable symptom alleviation, a brief, 4-week HT protocol may represent a more time- and cost-efficient choice for rapidly alleviating negative emotions in campus mental health settings.

However, to fundamentally enhance an individual's sense of life meaning and reduce the risk of extreme behaviors like suicide or self-harm, HT programs comprising 8 or more sessions are necessary; in practice, additional HT activities should be designed to meet program needs.

### 4.4 Limitations

This study has several limitations that warrant special attention:

This study only examined HT programs lasting 4 or 8 weeks, without further investigation into activities shorter than 4 weeks or longer than 8 weeks. Whether the immediate effects diminish beyond 8 weeks, or whether shorter programs (less than 4 weeks) can still influence depression and anxiety, remains to be explored. As research progresses, more combinations of duration and frequency should be established to investigate the “dose-response” relationship of HT.

The study sample size was relatively small, and some indicators' data did not conform to normal distribution, which to some extent undermined the generalizability of the statistical results. Importantly, the overall comparison of immediate effects (Table 2, upper panel) is presented as a descriptive summary of median effect sizes rather than a formal statistical test, due to the repeated-measures structure of sessions within participants. Formal between-group inferences rely on the session-level comparisons shown in Figure 3. Until larger parametric studies are conducted, these between-group findings should be viewed as preliminary. Although the Mann-Whitney *U* test showed no statistically significant differences between the two groups in terms of gender or academic level distribution, the proportion of females in the short-duration group was higher than that in the long-duration group. Previous research indicates that females have a high level of acceptance for HT activities (Kim and Park, 2018), which may have elevated the recovery effects observed in the short-duration group.

The between-group design comparing separate 4-week and 8-week cohorts—while methodologically appropriate for avoiding repeated-testing effects in a within-subject design—introduced inevitable inter-individual variability that may have confounded the estimated duration effects. This limits the strength of causal inference specifically regarding the differential impact of intervention length. To address this, future research could employ a waitlist control design or a crossover design with adequate washout periods, which would help disentangle duration effects from individual differences and strengthen the evidence base.

The extended duration of this study may have been influenced by environmental factors such as lighting, temperature, humidity, and participants' life events during the follow-up period.

Physiological mechanisms proposed in the discussion (e.g., parasympathetic activation) were inferred primarily from salivary cortisol changes. The absence of direct autonomic nervous system measures (e.g., heart rate variability, skin conductance) limits the strength of mechanistic claims. Multi-modal physiological assessments are recommended in future work.

Additionally, the analysis of immediate effects treated the 12 session-level pre-post measurements as independent observations, which does not account for within-subject correlations. This approach may underestimate standard errors and overstate the precision of effect size estimates. Although this choice was driven by the modest sample size and the non-parametric framework of the study, future investigations should employ mixed-effects models or generalized estimating equations (GEE) to appropriately model the repeated-measures structure and to confirm the stability of the immediate restorative effects reported here.

In summary, although this study provides initial evidence for the differential time-dose effects of HT, more rigorous experimental designs—including larger sample sizes, stratified randomization, alternative control conditions (e.g., waitlist controls, pure nature exposure), and possibly crossover or longitudinal within-subject designs — are needed to confirm and extend these findings.

## 5 Conclusions

Results indicate that HT induces significant and stable immediate psychological recovery. A single session significantly improves mood accompanied by a physiological decrease in salivary cortisol, and this effect does not diminish with extended intervention duration. Regarding cumulative effects, both the short-duration (4-week) and long-duration (8-week) programs produced within-group reductions in anxiety and depressive symptoms, although the between-group difference in effect sizes did not reach statistical significance. Given the comparable symptom alleviation achieved with less time and resources, a short-duration HT protocol may represent a more time- and cost-efficient option for transiently alleviating negative emotions in campus mental health services. However, longer-duration interventions proved crucial for fostering a stable and enduring enhancement of the deep psychological structure of “Meaning in Life.”

These findings systematically validate the effectiveness of HT in promoting college students' mental health from a temporal perspective, providing robust theoretical support for integrating this approach into campus mental health systems and expanding diverse intervention pathways.

Simultaneously, the study systematically reveals the “dose-response” relationship inherent in HT as a “green prescription” across temporal dimensions. It further identifies variations in this relationship across different outcome measures, thereby providing refined guidance for designing HT duration strategies tailored to specific intervention objectives.

At the mechanism level, it confirms that “establishing a life connection between humans and plants, perceiving and comprehending the meaning of life” is the core mechanism through which HT exerts psychological benefits and achieves sustained effects. Follow-up assessments further reveal the maintenance patterns of intervention effects, facilitating a more objective evaluation of its long-duration value and applicability boundaries.

Furthermore, this study innovatively distinguishes intervention effects into two temporal dimensions—“immediate effects” and “cumulative effects”—thereby more precisely delineating the influence of intervention duration on psychological benefits. This provides clearer directional guidance for subsequent research and practice.

## Data Availability

The original contributions presented in the study are included in the article/supplementary material, further inquiries can be directed to the corresponding author.
